# Carbon Dioxide Laser Ablation for the Treatment of a Rare Case of Acquired Lymphangioma Circumscriptum of the Vulva in an Adult Patient

**DOI:** 10.1155/crdm/9927730

**Published:** 2025-12-12

**Authors:** Harriet Rothschild, Sara McKinney

**Affiliations:** ^1^ Department of Obstetrics, Gynecology, and Reproductive Sciences, University of California, San Diego, California, USA, berkeley.edu

**Keywords:** CO_2_ laser, lymphangioma, lymphangioma circumscriptum, vulva

## Abstract

Lymphangioma circumscriptum is a rare condition that usually affects the mouth mucosa, tongue, axilla, groin, and proximal arms and legs. Vulvar involvement is uncommon and understudied, with no standardized treatment guidelines available. Lymphangioma circumscriptum is characterized by benign dilation of lymphatic channels in the skin and subcutaneous tissues, resulting in weeping and painful vesicular‐appearing growths. In this report, we present a unique case of a 50‐year‐old female with acquired lymphangioma circumscriptum of the vulva complicated by recurrent, painful vulvar papules. She elected for CO_2_ laser ablation treatment with successful cosmetic and symptomatic results. Despite the limited research and treatments available for lymphangioma circumscriptum, vulvar laser ablation appears to be a potentially safe and effective option.

## 1. Introduction

Lymphangioma circumscriptum (LC) is a rare lymphatic malformation that, although benign, can have a substantial impact on quality of life due to pain and cosmetic appearance [1]. LC is characterized by dilation of lymphatic vessels in the skin and subcutaneous tissue that do not communicate to the normal lymphatics [2]. These verrucous lesions can be painful, weep fluid, or blood, and delay of diagnosis is common as the lesions are often mistaken for warts [2]. LC generally occurs in areas of the body with dense lymphatic vasculature such as the axilla, groin, buttock, mouth, tongue, and less commonly the vulva [3, 4]. The etiology of congenital vulvar LC, defined by the symptoms occurring before 5 years of age, is unknown with only a few cases reported in the literature [3, 5]. Acquired LC, on the other hand, has a comparatively higher incidence and develops later in life [3]. The acquired form is associated with damage to lymphatic vessels such as direct blunt trauma, surgery, or radiation therapy [3, 4, 6]. Rarely, acquired LC of the vulva can be a sequela of gynecologic oncology procedures such as radical hysterectomy, pelvic lymph node dissection, or pelvic radiation [7].

In addition to affecting quality of life due to pain, itching, and discharge, vulvar LC can be cosmetically disfiguring and cause psychological distress [8]. Patients may struggle to achieve a diagnosis due to late presentation to care secondary to social stigma [8]. In addition, LC of the vulva is often misdiagnosed in clinic because it mimics other gynecologic conditions including genital warts, molluscum contagiosum, or herpes zoster [3]. Biopsy is often necessary to confirm diagnosis, which is an additional barrier to care for some patients.

There is no consensus or standard of care for treatment of either congenital or acquired vulvar LC. Surgery is the most common approach and while it offers definitive management, surgical excision carries greater morbidity, risk of complications, and worse cosmetic outcomes [3]. Here, we present an alternative treatment modality to surgical excision in a case of acquired vulvar LC treated with 10,600‐nm CO_2_ laser ablation with excellent results.

## 2. Case Presentation

**Figure 1:**
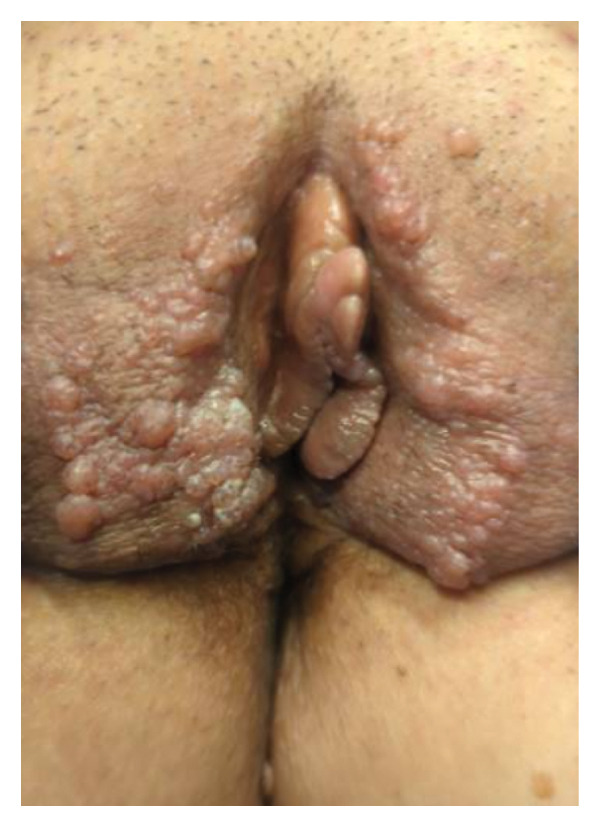
Initial presentation of large verrucous‐like lesions covering the labia majora, labia minora, and clitoral hood representing vulvar lymphangioma circumscriptum.

**Figure 2:**
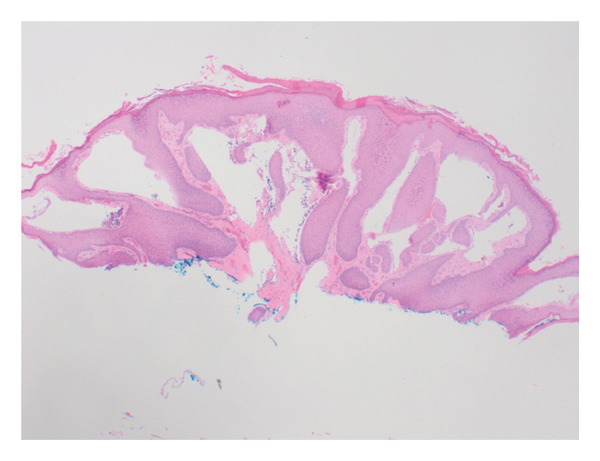
Histopathological section of the lesion showing typical features of lymphangioma circumscriptum with hyperkeratosis and dilated lymphatic channels in superficial dermis (H and E). Magnification 10x.

A 50‐year‐old woman with a history of T‐cell lymphoma presented to obstetrics and gynecology clinic with multiple painful weeping wart‐like lesions along her vulva, involving the labia majora, labia minora, clitoris, and mons pubis. According to the patient, the lesions developed two years after an excisional biopsy of a right inguinal lymph node for her lymphoma workup. On examination, bilateral labia majora were notable for multiple serous‐like papules. Smaller papule‐like lesions were also noted on the bilateral labia minora and a firm mass on the clitoral hood (Figure 1). The patient had previously been seen by a dermatologist with a differential diagnosis of hidradenitis suppurativa, dermatitis herpetiformis, genital warts, molluscum contagiosum, herpes zoster, or vulvar neoplasia. Routine blood examination was negative for Hepatitis C, HIV, and syphilis. Biopsy was performed for histopathological evaluation, which revealed hyperkeratosis and dilated lymphatic channels in the superficial dermis suggestive of LC (Figure 2).

**Figure 3:**
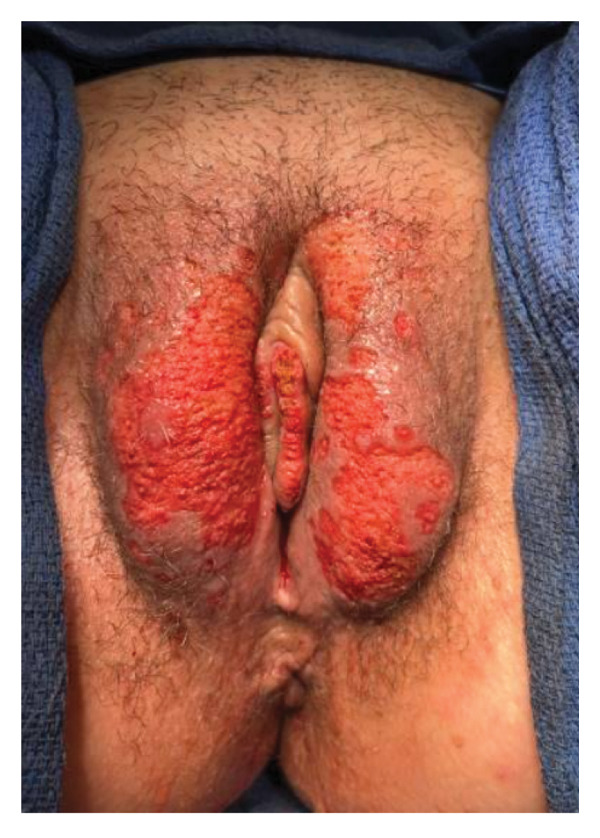
Vulva after immediately completing 10,600 nm CO_2_ laser treatment to a depth of 3 mm.

**Figure 4:**
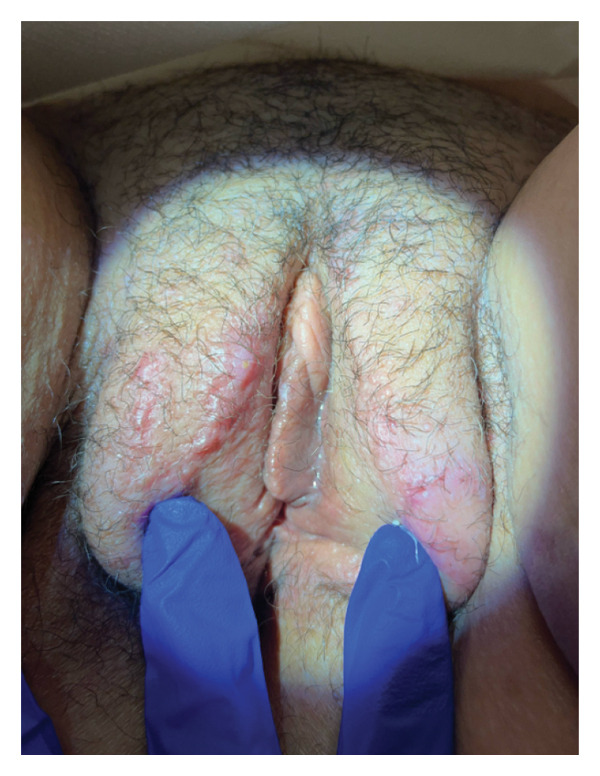
Vulva healed 8 weeks postoperatively.

The patient was counseled on available treatment options, which included topical rapamycin (sirolimus), sclerotherapy, radiotherapy, and vulvar laser ablation. She elected for surgical treatment with the AcuPulse DUO 10,600‐nm CO_2_ laser at 10 Watts with ablation to a depth of 3 mm (Figure 3). After the procedure, Silvadene cream was applied, and the patient was instructed to continue application for one month in addition to daily warm soaks. On postoperative Day 3, the patient developed an uncomplicated lower extremity cellulitis unrelated to the procedure that was treated with a short course of antibiotics. At follow‐up eight weeks postoperatively, the patient was very satisfied with the cosmetic results and resolution of the previously frequent weeping lesions (Figure 4). She had minimal evidence of recurrence. Future spot treatments with CO_2_ laser may be necessary to continue to maintain remission of disease.

## 3. Discussion

Lymphangiomas are congenital malformations of the lymphatic system that usually involve skin and subcutaneous tissue [9]. Lymphangioma can occur at any age, but majority are seen in children. They can be divided into superficial (LC), deep (lymphangioma cavernosum), and cystic (cystic hygroma) varieties. LC is a distinct form of lymphangioma that is localized to the skin and subcutaneous tissue. It was first described by Fox and Fox in 1879 [9]. Clinically, it manifests as pseudovesicles on the skin with areas of hyperkeratosis, giving it a papular and warty appearance [4]. The papules contain a clear, yellow, or pink‐colored fluid depending on the extent of blood content [4]. Furthermore, oozing, weeping, bleeding, itching, and general discomfort are symptoms commonly associated with these lesions [10].

The exact etiology of both congenital and acquired LC is unknown. However, various growth factors such as vascular endothelial growth Factor C (VEGF‐C) and VEGF‐D and their receptors on the lymphatic endothelial cells may have a role in the development of LC [11]. Acquired lymphangioma, which can occur at any age, generally develops secondary to chronic obstruction of lymphatic ducts due to physical injury, autoimmune conditions, infection, or iatrogenic procedures [12]. The most common causes of LC of the vulva are pelvic surgery, radiation therapy, autoimmune conditions such as ulcerative colitis, and infection [13]. Complications of LC of the vulva include cosmetic concerns, vulvar pain, recurrent cellulitis, and psychosexual dysfunction [14]. Furthermore, given the rarity of this condition, delay in diagnosis is not uncommon, as was experienced by this patient. Malignant transformation of LC is rare and usually a sequela of pelvic radiation therapy [9].

Surgical excision with vulvectomy has been the traditional treatment of LC; however, it has fallen out of favor and should only be considered after alternative treatment failures. Vulvectomy carries high morbidity and risk of early recurrence, wound complications, and poor cosmetic outcomes [15]. Medical management includes topical sirolimus, with the proposed mechanism of action to decrease VEGF, which as previously mentioned is thought to be involved in the lymphangiogenesis of LC [16]. Alternatively, vaporization with a CO_2_ laser is a recent recommendation for LC of the vulva and is suggested to yield satisfactory cosmetic and lasting results [15]. Topical sirolimus can be utilized after ablative treatment to prevent recurrence [17]. Here, we have shown that CO_2_ laser treatment can be used as an initial treatment modality in LC, as it is well tolerated, has minimal complications, and leads to excellent cosmetic and symptomatic outcomes. In conclusion, CO_2_ laser ablation is a safe and effective treatment for symptomatic LC, although continued spot treatment may be necessary.

## Consent

The patient provided written informed consent for publication of this case report and accompanying images, in accordance with the journal’s policy.

## Conflicts of Interest

The authors declare no conflicts of interest.

## Funding

The authors received no specific funding for this work.

## Data Availability

The data that support the findings of this study are available on request from the corresponding author. The data are not publicly available due to privacy or ethical restrictions.
